# Diagnostic and prognostic value of serum lactate dehydrogenase and amylase in severe acute pancreatitis: A clinical biochemistry-based evaluation

**DOI:** 10.5937/jomb0-63403

**Published:** 2026-05-22

**Authors:** Jie Zheng, Jin Wang, Yinong Zhou, Tingting Yu

**Affiliations:** 1 The Quzhou Affiliated Hospital of Wenzhou Medical University, Department of Pancreatology, Quzhou People's Hospital, Quzhou, China; 2 The Quzhou Affiliated Hospital of Wenzhou Medical University, Quzhou People's Hospital, Department of Cardiac Surgery, Quzhou, China

**Keywords:** lactate dehydrogenase, amylase, biochemical markers, severe acute pancreatitis, prognosis, ROC analysis, laktat-dehidrogenaza, amilaza, biohemijski markeri, teški akutni pankreatitis, prognoza, ROC analiza

## Abstract

**Background:**

Severe acute pancreatitis (SAP) is characterised by extensive acinar-cell injury, metabolic dysregulation, and systemic inflammatory activation. Reliable biochemical markers for early risk stratification remain limited. Lactate dehydrogenase (LDH) and amylase (AMY), routinely measured enzymes reflecting cellular damage and pancreatic exocrine dysfunction, may provide prognostic value. To evaluate the diagnostic performance of serum LDH and AMY in identifying SAP and predicting short-term outcomes.

**Methods:**

This retrospective study included 200 patients with acute pancreatitis. Serum LDH and AMY were measured on admission using standardised clinical chemistry assays with internal quality control. Patients were categorised as MAP, MSAP, or SAP, and SAP cases were further stratified into goodand poor-prognosis 2-week groups. Logistic regression and ROC analyses were used to determine the predictive value of LDH and AMY.

## Abstract (sr)

**Uvod:**

Teški akutni pankreatitis (SAP) karakterišu opsežno oštećenje acinarnih ćelija, metabolička disregulacija i sistemska inflamatorna aktivacija. Pouzdani biohemijski markeri za ranu stratifikaciju rizika su i dalje ograničeni. Laktat-dehidrogenaza (LDH) i amilaza (AMY), rutinski mereni enzimi koji odražavaju ćelijsko oštećenje i egzokrinu disfunkciju pankreasa, mogu da imaju prognostičku vrednost. Cilj ove studije bio je da se proceni dijagnostička efikasnost serumskih LDH i AMY u identifikaciji SAP i predviđanju kratkoročnih ishoda.

**Metode:**

Ova retrospektivna studija je obuhvatila 200 pacijenata sa akutnim pankreatitisom. Serumski nivoi LDH i AMY određeni su pri prijemu korišćenjem standardizovanih kliničko-hemijskih metoda sa internom kontrolom kvaliteta. Pacijenti su klasifikovani kao MAP, MSAP ili SAP, a slučajevi SAP su dodatno stratifikovani u grupe sa dobrom i lošom dvonedeljnom prognozom. Za procenu prediktivne vrednosti LDH i AMY korišćene su logistička regresija i ROC analize.

**Rezultati:**

Nivoi LDH i AMY su značajno rasli sa težinom bolesti (P&lt;0,05). Oba biomarkera bila su nezavisni prediktori SAP (LDH OR=1,644; AMY OR=1,670) i loše prognoze (LDH OR=1,684; AMY OR=1,730). Kombinovana upotreba LDH+AMY pokazala je superiornu prediktivnu tačnost za SAP (AUC=0,854) i kratkoročnu lošu prognozu (AUC=0,884), nadmašujući pojedinačne markere.

**Zaključak:**

Serumska LDH i AMY predstavljaju klinički značajne biohemijske pokazatelje za ranu identifikaciju teškog akutnog pankreatitisa i predviđanje kratkoročnih ishoda. Njihova kombinovana primena značajno unapređuje dijagnostičku tačnost i podržava uključivanje u rane biohemijske modele procene rizika.

## Introduction

Acute pancreatitis (AP) is a common acute abdominal disorder marked by sudden inflammation of the pancreas, destruction of acinar cells, and subsequent activation of digestive enzymes that initiate a cascade of local and systemic pathological responses [1]. As inflammation progresses, the disease may extend beyond the pancreas, leading to systemic inflammatory response syndrome and, in severe cases, multiple organ dysfunction syndrome. According to the revised Atlanta classification, AP is categorised into mild acute pancreatitis (MAP), moderately severe acute pancreatitis (MSAP), and severe acute pancreatitis (SAP). SAP is characterised by persistent organ failure and remains associated with substantial morbidity, mortality, and resource utilisation, underscoring the importance of early identification and intervention [2][3][4][5].

Metabolic disturbances, particularly involving glucose and lipid homeostasis, play a key role in the development and progression of AP Individuals with impaired glucose tolerance exhibit a heightened risk of AP onset and often present with more complicated disease trajectories compared with those with normal metabolic function [6]. In recent years, attention has increasingly focused on biochemical indicators reflective of underlying tissue injury and metabolic imbalance. Lactate dehydrogenase (LDH), a pivotal enzyme in glycolysis that catalyses the interconversion of lactate and pyruvate, is frequently elevated in conditions involving cellular damage and hypoxia, and several studies have linked LDH abnormalities to the occurrence and severity of AP [7]. Similarly, amylase (AMY), a key digestive enzyme involved in carbohydrate metabolism, remains an important biochemical marker in AP evaluation and is closely associated with acinar-cell injury and pancreatic exocrine dysfunction [8].

Despite widespread clinical use of these biochemical parameters, highly specific and sensitive early assessment tools for predicting SAP remain limited. Given the mechanistic relevance of LDH and AMY to metabolic dysregulation, cellular injury, and pancreatic enzyme activation, evaluating their diagnostic and prognostic potential may enhance early recognition of high-risk patients. Therefore, this study examines the expression levels of LDH and AMY across AP severity groups and investigates their value in predicting short-term prognosis in SAP, intending to provide a practical biochemical reference for early clinical risk stratification.

## Materials and methods

### General information

A retrospective study was conducted on 200 AP patients admitted to our hospital between September 2023 and September 2025. Age range: 42-75 years, mean (57.63±10.23) years; Gender: 110 males (55.00%), 90 females (45.00%).

Inclusion Criteria: ①Met AP diagnostic criteria per the »Expert Consensus on Emergency Diagnosis and Treatment of Acute Pancreatitis« [9]: sudden abdominal pain with or without nausea/vomiting, AMY and/or lipase >3 times the upper limit of normal, confirmed by imaging showing pancreatic edema, necrosis, or peri-pancreatic effusion; ②Age ≥18 years; ③Time from onset to admission ≤24 hours; ④Complete clinical data.

Exclusion Criteria: ①Complicated with pancreatic cancer or chronic pancreatitis; ②Pre-existing or other aetiology-induced organ failure; ③Pregnancy or lactation; ④Complicated with autoimmune diseases.

### Methods

### Grouping methods

AP patients were stratified into MAP (n = 52), MSAP (n = 65), and SAP (n=83) groups based on the Revised Atlanta Classification (2012) [10]. Organ failure was defined and assessed using the Modified Marshall Scoring System, with a score ≥2 in any organ system considered indicative of organ failure; MSAP included patients with transient organ failure (≤48 hours) and/or complications; MAP included patients without these features.

Pleural effusion was assessed by contrast-enhanced computed tomography (CT) scan performed within 48 hours of admission.

Given the high early mortality in SAP, short-term prognosis was defined by clinical status at two weeks. A poor prognosis was defined as the occurrence of any of the following within 14 days of admission: 1) In-hospital mortality; 2) Persistent organ failure (Modified Marshall score ≥2 in any organ system for >48 hours); or 3) The need for surgical or percutaneous intervention for pancreatic complications (e.g., necrosectomy, drainage). A good prognosis was defined as survival without persistent organ failure or the need for intervention.

### Data collection

Demographic and clinical variables were collected at admission, including age, gender, body mass index (BMI), comorbidities (diabetes, hypertension, coronary heart disease), smoking and alcohol consumption history, and the presumed aetiology of AP The Bedside Index for Severity in Acute Pancreatitis (BISAP) score was calculated for each patient based on five components: blood urea nitrogen (BUN) >8.9 mmol/L, impaired mental status, presence of systemic inflammatory response syndrome (SIRS), age >60 years, and pleural effusion on imaging. A BISAP score ≥3 was considered indicative of a high risk for SAP progression [11]. All clinical data were obtained from the hospital's electronic medical record system and independently verified by two investigators to ensure accuracy and completeness.

### Serum LDH and AMY measurement

Fasting venous blood samples (5 mL) were collected from all patients within 24 hours of admission, before any major resuscitation or surgical intervention. After allowing the samples to clot for 20-30 minutes at room temperature, they were centrifuged at 3,500 rpm for 10 minutes. The resulting serum was promptly aliquoted into polypropylene tubes to avoid repeated freeze-thaw cycles, and all biochemical measurements were performed in the hospital's Clinical Biochemistry Laboratory under standardised internal quality control procedures.

The analyser automatically flagged potential analytic interferences from hemolysis, lipemia, or icterus. Samples with significant hemolysis (hemolysis index >100) were excluded from the analysis to ensure the accuracy of LDH measurements.

Serum LDH concentrations were determined using a fully automated biochemical analyser based on the lactate-to-pyruvate colourimetric enzymatic method. The LDH assay was performed on a Roche Cobas c702 analyser (Roche Diagnostics, Basel, Switzerland) using reagents from Shanghai Yaji Biological. The assay measures LDH activity by monitoring the rate of NAD+ reduction to NADH at 340 nm under strictly controlled conditions at 37°C, using a kinetic rate approach. Daily two-point calibration was performed using manufacturer-supplied standards, and the assay's analytical precision was continuously monitored through bilevel quality-control materials analysed twice per day, maintaining a coefficient of variation below 5%. The established reference interval was 120-250 U/L.

Serum AMY levels were assessed using an enzymatic rate method. The AMY assay was performed on a Beckman Coulter AU5800 analyser (Beckman Coulter, Brea, CA, USA) using reagents from Beijing Comac Biological. The analyser monitored absorbance at 405 nm at 37°C, and the rate of increase was proportional to amylase activity. Calibration curves were generated according to reagent-specific requirements, and quality assurance was maintained through routine analysis of low- and high-level control materials, with the analytical coefficient of variation consistently maintained below 3%. The established reference interval was 30-110 U/L.

### Statistical analysis

Data were analysed using the Statistical Package for Social Science (SPSS) 28.0 (IBM, Armonk, NY, USA). Categorical data are presented as n(%) and compared using the c^2^ test. Continuous data are presented as (x̄±s) and compared using t-tests or ANOVA. Multivariate logistic regression identified factors influencing SAP occurrence and poor prognosis. Multicollinearity among the independent variables was assessed using the Variance Inflation Factor (VIF). A VIF value greater than 10 was considered indicative of significant multicollinearity. Predictive value was assessed using ROC analysis. *P*<0.05 was considered statistically significant. The combined predictor was constructed using a multivariate logistic regression model that included both LDH and AMY as continuous variables. The model's predicted probability was used for the ROC analysis. Internal validation of the combined model was performed using 1000 bootstrap samples to obtain a bias-corrected AUC and 95% confidence interval. Model calibration was assessed using the Hosmer-Lemeshow goodness-of-fit test and by calculating the Brier score. A non-significant Hosmer-Lemeshow test (P>0.05) and a lower Brier score indicate better calibration. The DeLong test was used to compare the areas under the ROC curves (AUCs) between different models.

## Results

### Comparison of baseline data, LDH, and AMY in AP patients by severity

As shown in Table 1, significant differences were found among the three groups in BISAP score, SIRS incidence, WBC, respiratory rate, heart rate, BUN, AMY, and LDH levels (P<0.05). No differences were found in other baseline characteristics (P>0.05).

**Table 1:** Comparison of baseline data, LDH, and AMY in AP patients by severity [x̄ ± s, n (%)].

Baseline data	MAP group<br>(*n* = 52)	MSAP group<br>(*n* = 65)	SAP group<br>(*n* = 83)	c^2^/*F*	*P*
Male	30 (57.69)	38 (58.46)	42 (50.60)	1.116	0.572
Female	22 (42.31)	27 (41.54)	41 (49.40)		
Age (years)	55.94±8.12	57.63±12.23	58.76±10.35	1.153	0.318
BMI (kg/m^2^)	22.75±1.08	22.51±1.31	22.97±1.25	2.558	0.080
Diabetes	10 (19.23)	12 (18.46)	19 (22.89)	0.508	0.776
Coronary Heart Disease	5 (9.62)	8 (12.31)	10 (12.05)	0.248	0.884
Hypertension	14 (26.92)	17 (26.15)	25 (30.12)	0.325	0.850
Smoking history	12 (23.08)	18 (27.69)	22 (26.51)	0.339	0.844
Alcohol history	8 (15.38)	10 (15.38)	15 (18.07)	0.255	0.880
Etiology biliary	25 (48.08)	30 (46.15)	35 (42.17)	0.503	0.778
Non-biliary	27 (51.92)	35 (53.85)	48 (57.83)		
Pleural effusion	10 (19.23)	18 (27.69)	31 (37.35)	5.198	0.074
BISAP Score (points)	2.18±0.19	2.49±0.24	3.21±0.45	171.353	0.000
SIRS	15 (28.85)	19 (29.23)	40 (48.19)	7.627	0.022
WBC (×10^9^/L)	12.29±1.94	13.87±2.11	15.76±2.05	47.769	0.000
Respiratory rate (breaths/<br>min)	20.39±1.62	23.41±2.37	26.37±1.74	154.365	0.000
Heart rate (beats/min)	96.35±10.51	107.56±9.26	114.52±7.85	64.279	0.000
BUN (mmol/L)	7.15±1.04	8.21±1.48	8.97±1.21	33.120	0.000
AMY (U/L)	311.47±30.29	429.53±40.71	547.27±52.34	472.122	0.000
LDH (U/L)	185.23±46.32	221.67±55.63	264.18±43.47	43.814	0.000

### Multivariate logistic regression analysis of factors influencing SAP occurrence

Using disease severity as the dependent variable (SAP=1, MSAP/MAP=0) and SIRS (present=1, absent=0), BISAP score, WBC, respiratory rate, heart rate, BUN, AMY, and LDH (actual values) as independent variables, logistic regression showed that SIRS, BISAP score, WBC, respiratory rate, heart rate, AMY, and LDH were independent influencing factors for SAP occurrence (P<0.05) (Table 2).

**Table 2:** Multivariate logistic regression analysis of factors influencing SAP occurrence.

Variable	*β*	SE	Wald c^2^	*P*	*OR*	95% *CI*	VIF
Age	0.215	0.142	2.291	0.130	1.240	0.939-1.637	1.8
Impaired mental<br>status	0.398	0.267	2.223	0.136	1.489	0.882-2.515	1.2
SIRS	0.387	0.243	5.919	0.007	1.473	1.109-1.976	2.1
Pleural effusion	0.312	0.201	2.410	0.121	1.366	0.921-2.026	1.5
BUN	0.226	0.118	3.421	0.174	1.254	1.027-1.458	1.4
WBC	0.312	0.207	5.228	0.017	1.366	1.254-1.711	1.9
Respiratory rate	0.342	0.216	5.572	0.015	1.408	1.173-2.377	2.3
Heart rate	0.359	0.221	5.714	0.010	1.432	1.169-1.782	1.7
AMY	0.513	0.374	7.742	<0.001	1.670	1.314-1.987	1.6
LDH	0.497	0.308	7.429	<0.001	1.644	1.239-2.110	1.5

### Predictive value of LDH and AMY for SAP occurrence

**Figure 1:**
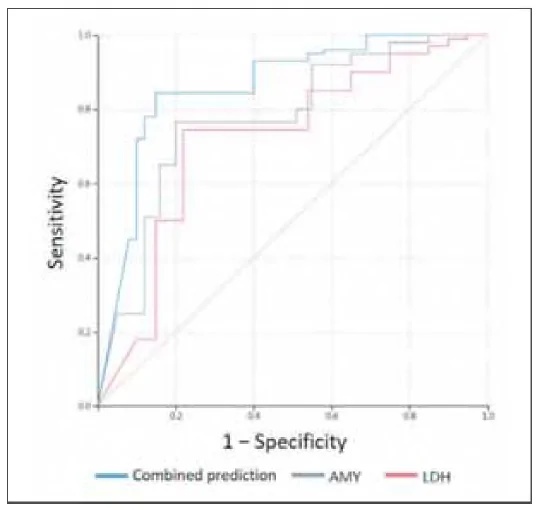
ROC curve of LDH and AMY for predicting the occurrence of SAP.

As shown in Figure 1 and Table 3, ROC analysis showed that the combined prediction of AMY and LDH for SAP occurrence had a sensitivity of 84.43%, specificity of 85.61%, and an AUC of 0.854, which was higher than either marker alone (*P*<0.05).

**Table 3:** Predictive value of LDH and AMY for SAP occurrence.

Variable	AUC	Cut-off value	95% CI	Sensitivity (%)	Specificity (%)	PPV (%)	NPV (%)
AMY	0.759	450 U/L	0.706-0.843	76.55	75.42	75.6	84.2
LDH	0.742	350 U/L	0.684-0.809	74.47	72.56	73.2	81.8
Combined	0.854	-	0.803-0.925	84.43	85.61	82.1	91.2

### For SAP occurrence

To assess the additive value of LDH and AMY beyond the BISAP score, we compared the predictive performance of BISAP alone (AUC = 0.812) with a model combining BI SAP, LDH, and AMY (AUC=0.901). The combined model demonstrated a significantly higher AUC (*P*<0.05), indicating improved discrimination.

### Comparison of baseline data, LDH, and AMY in SAP patients by short-term prognosis

As illustrated in Table 4, the poor-prognosis group had significantly higher BISAP scores, SIRS incidence, respiratory rate, AMY, and LDH levels than the good-prognosis group (*P*<0.05). No significant differences were found in other baseline data (*P*>0.05).

**Table 4:** Compa rison of baseline data, LDH, and AMY in SAP patients by short-term prognosis [x̄ ± s, n (%)].

Baseline data	Poor group<br>(*n*=30)	Good group<br>(*n* = 53)	c^2^/*t*	*P*
Male	17 (56.67)	25 (47.17)	0.691	0.406
Female	13 (43.33)	28 (52.83)		
Age (years)	58.14±9.78	59.35±10.22	0.526	0.600
BMI (kg/m^2^)	22.78±1.21	23.05±1.04	1.071	0.288
Diabetes	9 (30.00)	10 (18.87)	1.345	0.246
Coronary Heart Disease	4 (13.33)	6 (11.32)	0.006	0.936
Hypertension	10 (33.33)	15 (28.30)	0.230	0.631
Smoking history	10 (33.33)	12 (22.64)	1.124	0.289
Alcohol history	6 (20.00)	9 (16.98)	0.118	0.731
Etiology biliary	13 (43.33)	22 (41.51)	0.026	0.872
Non-biliary	17 (56.67)	31 (58.49)		
Pleural effusion	16 (53.33)	15 (28.30)	5.130	0.024
BISAP Score (points)	3.83±0.44	3.17±0.23	8.989	0.000
SIRS	20 (66.67)	20 (37.74)	6.422	0.011
WBC (×10^9^/L)	16.17±1.45	15.34±2.62	1.599	0.114
Respiratory rate (breaths/min)	30.29±3.17	24.36±2.71	9.002	0.000
Heart rate (beats/min)	119.25±16.24	112.68±13.84	1.950	0.055
BUN (mmol/L)	9.07±0.84	8.85±1.12	0.936	0.352
AMY (U/L)	684.73±110.21	519.36±84.67	7.650	0.000
LDH (U/L)	432.48±80.37	241.98±65.82	11.682	0.000

### Multivariate logistic regression analysis of factors influencing poor short-term prognosis in SAP patients

Using prognosis as the dependent variable (Poor=1, Good=0) and SIRS, BISAP score, respiratory rate, AMY, and LDH (actual values) as independent variables, logistic regression showed that BISAP score, SIRS, respiratory rate, AMY, and LDH were independent factors for poor short-term prognosis in SAP patients (*P*<0.05) (Table 5).

**Table 5:** Multivariate logistic regression analysis of factors influencing poor short-term prognosis in SAP patients.

Variable	b	SE	Wald c^2^	P	OR	95% CI	VIF
Age	0.198	0.154	1.654	0.198	1.219	0.901-1.650	1.7
Impaired mental status	0.412	0.278	2.195	0.139	1.510	0.875-2.605	1.3
Pleural effusion	0.325	0.212	2.348	0.125	1.384	0.913-2.098	1.6
SIRS	0.411	0.252	6.014	0.007	1.508	1.127-1.692	2.2
Respiratory rate	0.372	0.237	5.828	0.004	1.451	1.358-1.934	2.4
AMY	0.548	0.397	8.275	<0.001	1.730	1.293-2.735	1.7
LDH	0.521	0.384	7.958	<0.001	1.684	1.349-2.526	1.6

### Predictive value of LDH and AMY for poor short-term prognosis in SAP patients

**Figure 2:**
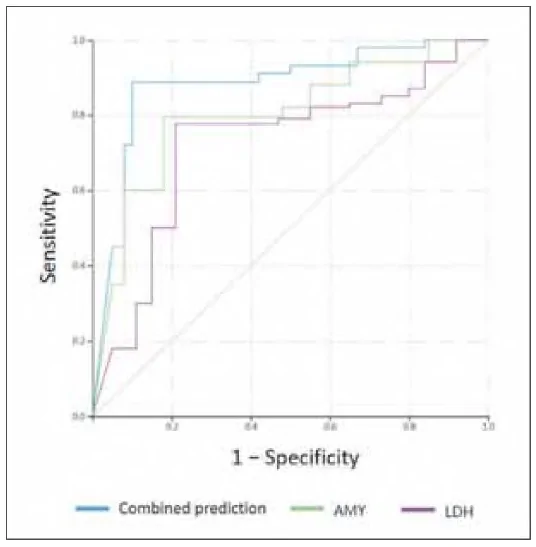
ROC curve of LDH and AMY in predicting short-term adverse prognosis in patients with severe acute pancreatitis (SAP).

As depicted in Figure 2 and Table 6, ROC analysis showed that the combined prediction of AMY and LDH for poor short-term prognosis had a sensitivity of 88.62%, specificity of 87.34%, and an AUC of 0.884, which was higher than either marker alone (*P*<0.05).

**Table 6:** Predictive value of LDH and AMY for poor short-term prognosis in SAP patients.

Variable	AUC	Cut-off value	95% CI	Sensitivity<br>(%)	Specificity<br>(%)	PPV<br>(%)	NPV<br>(%)
AMY	0.776	480 U/L	0.708-0.845	79.45	78.31	76.2	86.7
LDH	0.752	380 U/L	0.691-0.836	77.64	75.16	74.1	84.6
Combined	0.884	-	0.805-0.937	88.62	87.34	84.6	92.3

### Poor prognosis

Similarly, for predicting poor short-term prognosis in SAP patients, the combined model (BISAP + LDH + AMY) achieved an AUC of 0.923, which was significantly higher than the BISAP score alone (AUC=0.815,* P*<0.05).

## Discussion

The pathogenesis of severe acute pancreatitis (SAP) is multifactorial. It reflects the interplay between premature activation of pancreatic enzymes, autodigestion of pancreatic tissue, and a subsequent cascade of inflammatory and metabolic disturbances [12]. In clinical practice, early recognition of SAP remains challenging because commonly used scoring systems, such as BISAP although simple and widely validated, rely on multiple clinical variables that may lack biochemical specificity and are less sensitive during the early stages of disease progression [13]. Similarly, non-specific indicators such as white blood cell count, respiratory rate, and heart rate reflect systemic stress but do not directly capture biochemical injury processes that characterise SAP These limitations highlight the need for reliable biochemical markers that reflect underlying cellular damage, metabolic dysregulation, and inflammatory activation.

Lactate dehydrogenase (LDH), a key enzyme in aerobic glycolysis, plays a central role in energy metabolism and serves as a sensitive indicator of tissue injury. LDH catalyses the reversible interconversion of lactate and pyruvate and is released into the circulation when cellular integrity is compromised. In this study, LDH levels were significantly elevated in SAP patients and independently associated with both the occurrence of SAP and short-term adverse prognosis, consistent with findings reported by Xu Binbin et al. [14]. The biochemical relevance of LDH in SAP can be understood through several mechanistic pathways. Increased LDH activity reflects enhanced glycolytic flux and lactate accumulation, which can amplify inflammatory responses. Lactate derived from glycolysis promotes adipose tissue macrophage polarisation and enhances cytokine production, thereby contributing to systemic inflammatory response syndrome (SIRS) and multiorgan dysfunction [15]. Moreover, recent evidence suggests that glycolytic metabolites, including lactate, can modulate epigenetic processes such as histone lactylation, creating a positive feedback loop that drives inflammatory and metabolic dysregulation in pancreatic tissue [16]. Lactate may also bind to Prolyl Hydroxylase Domain 2 (PHD2), modulating hypoxia-inducible factor (HIF) stability and promoting inflammatory macrophage activation. These biochemical disruptions, coupled with impaired mitochondrial ATP generation, can lead to insufficient energy reserves for cellular repair, further exacerbating tissue injury in SAP. Persistent LDH elevation may therefore reflect ongoing cellular necrosis, systemic inflammatory propagation, and impaired organ function, explaining its association with poor short-term outcomes [17].

Amylase (AMY), a classical digestive enzyme involved in polysaccharide degradation, remains one of the earliest and most frequently measured biomarkers in suspected AP Serum AMY typically rises sharply following acinar cell injury, peaks within 24-48 hours, and gradually normalises within several days. This study demonstrated that AMY levels were significantly higher in SAP patients and independently associated with both disease severity and short-term prognosis, consistent with findings from Hu Yulong et al. [18]. The biochemical interpretation of AMY elevation extends beyond acinar-cell rupture. AMY participates in the acinar-islet-acinar metabolic feedback loop, regulating glucose homeostasis. Exogenous AMY in peri-islet tissue has been shown to delay glucose-stimulated insulin release, linking pancreatic exocrine injury to endocrine dysfunction [19]. In SAP, inflammatory cytokines may further increase AMY levels by promoting Caspase-1-dependent pyroptosis, a process that exacerbates acinar cell death and increases AMY leakage into the bloodstream [20].

Additionally, the uncontrolled release of AMY and other pancreatic enzymes contributes to intra-pancreatic trypsin activation, worsening inflammation, tissue necrosis, and systemic complications. AMY may also influence systemic glucose and energy metabolism by modulating glucose transporter activity, glycolytic enzyme function, and insulin secretion dynamics [21]. These metabolic and inflammatory interactions may help explain why higher AMY levels were observed in SAP patients with poor short-term prognosis.

A notable finding of this study is the superior diagnostic and prognostic performance of the combined LDH+AMY model compared with either biomarker alone. ROC analysis demonstrated that the combination improved sensitivity and specificity for predicting SAP occurrence and short-term poor outcomes. This enhanced performance likely arises from the complementary biochemical pathways reflected by the two markers. LDH captures the degree of cellular injury, glycolytic stress, and lactate-driven inflammatory activation, whereas AMY reflects the extent of acinar-cell disruption, dysregulated exocrine secretion, and broader metabolic imbalance. Because SAP involves simultaneous biochemical disturbances across both metabolic and digestive pathways, integrating LDH and AMY provides a more comprehensive biochemical profile of disease severity. Using either marker alone risks missing important pathophysiological information, whereas their combined application reduces misclassification and improves early risk stratification.

From a clinical biochemistry perspective, LDH and AMY possess several advantages. Both assays are inexpensive, rapid, widely available, and analytically robust with well-established methodologies in routine laboratory practice. Based on the logistic regression model, a probability cut-off of 0.65 for the combined model (BISAP+lDh+AMY) can be used for risk stratification. Patients with a probability ≥0.65 are at high risk for SAP or poor prognosis and may benefit from more intensive monitoring. However, as this study was retrospective, potential selection and information biases cannot be excluded. Prospective, multicenter studies are needed to validate these findings and further refine biochemical prediction models incorporating LDH, AMY, and potentially additional emerging biomarkers.

## Conclusion

In summary, serum LDH and AMY are valuable biochemical indicators for evaluating SAP severity and predicting short-term clinical outcomes. Their combined use provides superior diagnostic and prognostic accuracy compared with either marker alone, reflecting complementary aspects of metabolic disturbance and acinar-cell injury. Given the retrospective design of this study, potential selection and information biases cannot be excluded. Future multicenter, prospective studies are warranted to validate these findings, identify additional laboratory biomarkers, and develop more refined biochemical prediction models for early risk stratification in SAP.

## Dodatak

### Acknowledgments

This study did not receive any funding.

### Ethical statement

This study was approved by the ethics committee of The Quzhou Affiliated Hospital of Wenzhou Medical University (Approval no. 2026-017). Signed written informed consents were obtained from the patients and/or guardians. This study was conducted in accordance with the Declaration of Helsinki.

### Conflict of interest statement

All the authors declare that they have no conflict of interest in this work.

## References

[1] Hong W, Zheng L, Lu Y, Qiu M, Yan Y, Basharat Z, et al (2022). Non-linear correlation between amylase day 2 to day 1 ratio and incidence of severe acute pancreatitis. Front Cell Infect Microbiol.

[2] Shuanglian Y, Huiling Z, Xunting L, Yifang D, Yufen L, Shanshan X, et al (2023). Establishment and validation of early prediction model for hypertriglyceridemic severe acute pancreatitis. Lipids Health Dis.

[3] Zhou W, Liu Q, Wang Z, Yao L, Chen J, Yang X (2024). Analysis of the clinical profile and treatment efficiency of hyperlipidemic acute pancreatitis. Lipids Health Dis.

[4] Chen S, Song X, Lu J, Liang J, Ouyang H, Zheng W, Chen J, Yin Z, Li H, Zhou Y (2024). Application of alkaline phosphatase-to-hemoglobin and lactate dehydrogenase-to-hemoglobin ratios as novel noninvasive indices for predicting severe acute pancreatitis in patients. PLoS One.

[5] Bush N, Rana S S (2022). Ascites in Acute Pancreatitis: Clinical Implications and Management. Digest Dis Sci.

[6] Barbieri J S, Riggio J M, Jaffe R (2016). Amylase testing for abdominal pain and suspected acute pancreatitis. J Hosp Med.

[7] Ismail O Z, Bhayana V (2017). Lipase or amylase for the diagnosis of acute pancreatitis?. Clin Biochem.

[8] Yang D, Lu H, Liu Y, Li M, Hu W, Zhou Z (2022). Development and validation of a prediction model for moderately severe and severe acute pancreatitis in pregnancy. World J Gastroenterol.

[9] Rao J, Li J, Wu Y, Lai T, Zhou Z, Gong Y, Xia Y, Luo L, Xia L, Cai W, Huang W, Zhu Y, He W (2025). Ascites characteristics in acute pancreatitis: A prognostic indicator of organ failure and mortality. World J Gastroenterol.

[10] Colvin S D, Smith E N, Morgan D E, Porter K K (2020). Acute pancreatitis: an update on the revised Atlanta classification. Abdom Radiol (NY).

[11] Wang Z, Liu Y, Zhang X, Wang C, Tian J, Zhao H, Tian Q, Qu H (2025). Construction of a nomogram for hypertriglyceridemic severe acute pancreatitis that includes metabolic indexes. Lipids Health Dis.

[12] Tian F, Li H, Wang L, Li B, Aibibula M, Zhao H, Feng N, Lv J, Zhang G, Ma X (2020). The diagnostic value of serum C-reactive protein, procalcitonin, interleukin-6 and lactate dehydrogenase in patients with severe acute pancreatitis. Clinica Chimica Acta.

[13] Lu G, Pan Y, Kayoumu A, Zhang L, Yin T, Tong Z, Li B, Xiao W, Ding Y, Li W (2017). Indomethacin inhabits the NLRP3 inflammasome pathway and protects severe acute pancreatitis in mice. Biochem Biophys Res Commun.

[14] Furey C, Buxbaum J, Chambliss A B (2020). A review of biomarker utilization in the diagnosis and management of acute pancreatitis reveals amylase ordering is favored in patients requiring laparoscopic cholecystectomy. Clin Biochem.

[15] Morton A (2022). Review article: Diagnosing acute pancreatitis in diabetes mellitus. Emergency Medicine Australasia: EMA.

[16] Wang X, Zhang Y (2024). Diagnostic Value of Serum Amylase and Coagulation Function Indices in Distinguishing Acute Pancreatitis from Aortic Dissection. Clin Lab.

[17] Boggio V, Gonzalez C D, Zotta E, Ropolo A, Vaccaro M I (2025). VMP1 Constitutive Expression in Mice Dampens Pancreatic and Systemic Histopathological Damage in an Experimental Model of Severe Acute Pancreatitis. International Journal of Molecular Sciences.

[18] Wang B, Huang D, Cao C, Gong Y (2023). Insect α-Amylases and Their Application in Pest Management. Molecules.

[19] Azzopardi E, Lloyd C, Teixeira S R, Conlan R, Whitaker I S (2016). Clinical applications of amylase: Novel perspectives. Surgery.

[20] Kumar V, Barkoudah E, Souza D A T, Jin D X, McNabb-baltar J (2021). Clinical course and outcome among patients with acute pancreatitis and COVID-19. Eur J Gastroenterol Hepatol.

[21] Bengi G, Çelik İ, Dolu S, Önem S, Soytürk M, Rendeci S, Topalak Ö, Akpinar H (2025). Neutrophil-Lymphocyte Ratio and LDH/Albumin Ratio as Biomarkers for Severity and Mortality in Acute Pancreatitis. Official Journal of Turkish Society of Gastroenterology.

